# Supplementary material for: Alarming Antibiotic Resistance of Lactobacilli Isolated from Probiotic Preparations and Dietary Supplements
Source: Antibiotics (Basel). 2022 Nov 5;11(11):1557. doi: 10.3390/antibiotics11111557 (PMC9686474; doi:10.3390/antibiotics11111557)

Supplementary Material

Table S1. Standards for interpreting of inhibition zone diameters for antibiotics used in this study

No.	Antibiotic	Mechanism of action	Concen-tration, μg/disc	Zone of inhibition (mm) [a]			Reference
				R	MS	S	
1	Ampicillin	Cell wall synthesis inhibitors	10	≤ 12	13-15	≥ 16	[44]
2	Amoxicillin		20	≤ 13	14-16	≥ 17	[45]
3	Cefepim		30	≤14	15-19	≥20	[15]
4	Cefoperazone		75	≤14	15-19	≥20	
5	Cefoperazone / sulbactam		50/50	≤14	15-19	≥20	[45]
6	Cefazolin		30	≤14	15-19	≥20	[15]
7	Cefotaxime		30	≤14	15-22	≥23	[45]
8	Ceftriaxone		30	≤14	15-19	≥20	[15]
9	Ceftazidime		30	≤14	15-19	≥20	
10	Ertapenem		10	≤14	15-19	≥20	
11	Imipenem		10	≤ 13	14-15	≥ 16	[45]
12	Meropenem		10	≤14	15-19	≥20	[15]
13	Vancomycin		30	≤ 14	15-16	≥ 17	[45]
14	Ciprofloxacin	DNA gyrase inhibitor	5	≤ 13	14-18	≥ 19	
15	Norfloxacin		10	≤ 13	14-18	≥ 19	
16	Amikacin	Protein synthesis inhibitors	30	≤ 15	16-17	≥ 18	
17	Chloramphenicol		30	≤ 13	14-17	≥ 18	
18	Clarithromycin		15	≤14	15-19	≥20	[15]
19	Erythromycin		15	≤13	14-17	≥18	[45]
20	Linezolid		30	≤14	15-19	≥20	[15]
21	Tetracycline		30	≤ 14	15-18	≥ 19	[45]

[a] Ranges of zone of inhibition diameters exhibited by bacteria considered susceptible (S), moderately susceptible (MS), or resistant (R) to each antibiotic are shown.

Table S2. Specific primers and conditions for polymerase chain reaction (PCR) detection of antibiotic resistance genes

No.	Target gene	Determining resistance to	Mechanism of resistance	Primer sequence (5′→3′)	Amplicon size (bp)	Annealing temperature (◦C)	Reference
1	*erm*B	Erythromycin	Ribosomal methylation	CATTTAACGACGAAACTGGC<br>GGAACATCTGTGGTATGGCG	425	60	[46]
2	*tet*M	Tetracycline	Ribosomal protection proteins	GGTGAACATCATAGACACGC<br>CTTGTTCGAGTTCCAATGC	401	58	[47]
3	*tet*K		Efflux	TTATGGTGGTTGTAGCTAGAAA<br>AAAGGGTTAGAAACTCTTGAAA	348	55	[48]
4	*gyr*A	Ciprofloxacin	Modification of the GyrA subunit of DNA gyrase	GAYTATGCWATGTCAGTTATTGT<br>GGAATRTTRGAYGTCATACCAAC	286	45	[49]
5	*par*C		Modification of the ParC subunit of topoisomerase IV	TATTCYAAATAYATCATTCARGA<br>GCYTCNGTATAACGCATMGCCG	286	50	[14]
7	*cat*	Chloramphenicol	Acetylation of the antibiotic (acetyltransferase)	TTAGGTTATTGGGATAAGTTA<br>GCATGRTAACCATCACAWAC	300	52	
8	*van*E	Vancomycin	Replacement of normal peptidoglycan precursors by modified precursors	TGTGGTATCGGAGCTGCAG<br>GTCGATTCTCGCTAATCC	513	52	[50]
9	*van*A			CATGAATAGAATAAAAGTTGCAATA<br>CCCCTTTAACGCTAATACGATCAA	1030	54	[51]
10	*van*X			TCGCGGTAGTCCCACCATTCGTT<br>AAATCATCGTTGACCTGCGTTAT	454	55	[32]
11	*bla*TEM	Cephalosporins	Extended-spectrum β-lactamase	ATCAGCAATAAACCAGC<br>CCCCGAAGAACGTTTTC	516	54	[52]
12	*bla*SHV			AGGATTGACTGCCTTTTTG<br>ATTTGCTGATTTCGCTCG	392	54	
13	*bla*OXA-1			ATATCTCTACTGTTGCATCTCC<br>AACCCTTCAAACCATCC	619	54	
14	*bla*VIM	Carbapenems	Metallo-β-lactamase	GTTTGGTCGCATATCGC<br>TCGTCATGAAAGTGCGT	175	50	[53]
15	*bla*IMP1			GCTAAAGATACTGAAAAATTAGT<br>TCATTTGTTAATTCAGATGCATA	174	50	

Table S3. The antibiotic susceptibility profile of *Lactobacillus* strains for erythromycin, chloramphenicol and tetracycline

No.	Strain	Erythromycin		Chloramphenicol		Tetracycline				
		**Zone of inhibition (mm)**	**Inter-pretation**	**Zone of inhibition (mm)**	**Inter-pretation**	**Zone of inhibition (mm)**	**Inter-pretation**	**MIC (µg/mL)**	**Break-point (EFSA, 2008)**	**Interpretation**
1	*L. plantarum* 8PA3	14.0±3.5	MS	19.0±3.0	S	26.0±4.0	S	2	32	S
2	*L. fermentum* Gm	14.0±0.5	MS	32.0±3.0	S	26.5±1.5	S	2	8	S
3	*L. plantarum* Ne	18.0±1.0	S	32.5±3.5	S	18.5±1.5	S	4	32	S
4	*L. plantarum* Ro-1	18.0±1.4	S	21.5±0.7	S	15.5±3.5	MS	32	32	S
5	*L. plantarum* Ro-2	18.5±0.7	S	22.0±2.8	S	18.5±0.7	S	16	32	S
6	*L. plantarum* Ro-5	19.5±0.7	S	20.5±0.7	S	16.5±0.7	MS	16	32	S
7	*L. plantarum* Ro-7	18.5±2.1	S	25.5±0.7	S	16.5±0.7	MS	16	32	S
8	*L. plantarum* Ro-8	18.5±0.7	S	23.5±2.1	S	17.5±0.7	MS	16	32	S
9	*L. plantarum* At-1	18.5±0.7	S	24.5±2.1	S	18.0±0.0	MS	16	32	S
10	*L. plantarum* At-2	19.0±2.8	S	29.0±1.4	S	19.0±2.8	S	8	32	S
11	*L. plantarum* At-3	20.0±0.0	S	25.5±2.1	S	18.0±2.8	MS	16	32	S
12	*L. plantarum* Ls	17.5±0.7	S	26.5±2.1	S	15.5±2.1	MS	16	32	S
13	*L. paracasei* Ea-1	24.0±1.4	S	26.0±0.0	S	26.5±2.1	S	4	4	MS
14	*L. paracasei* Ea-2	18.5±6.4	S	25.5±0.7	S	28.0±0.0	S	8	4	R
15	*L. paracasei* Ea-3	25.5±0.7	S	25.0±0.0	S	27.5±0.7	S	8	4	R
16	*L. helveticus* Al-1	25.5±0.7	S	28.5±2.1	S	26.5±4.9	S	16	4	R
17	*L. helveticus* Al-2	27.5±0.7	S	31.0±1.4	S	30.5±0.7	S	16	4	R
18	*L. helveticus* Al-3	32.0±0.0	S	29.5±2.1	S	33.5±2.1	S	16	4	R
19	*L. helveticus* Al-4	30.0±1.4	S	25.0±0.0	S	33.5±0.7	S	16	4	R

Lactobacilli were classified either as susceptible (S), moderately susceptible (MS), or resistant (R) to each antibiotic based on zones of growth inhibition mentioned in [45] in the case of the disc diffusion method and breakpoint values defined in [43] in the case of MIC values of tetracycline determined by the broth microdilution method.

Figure S1. Representative MRS agar plates of bacteria recovered from probiotics and dietary supplements showing different colony morphologies and densities. Starting from the upper left sector cw sectors correspond to dilutions 10^{-1}, 10^{-4}, 10^{-6}, and 10^{-7}.

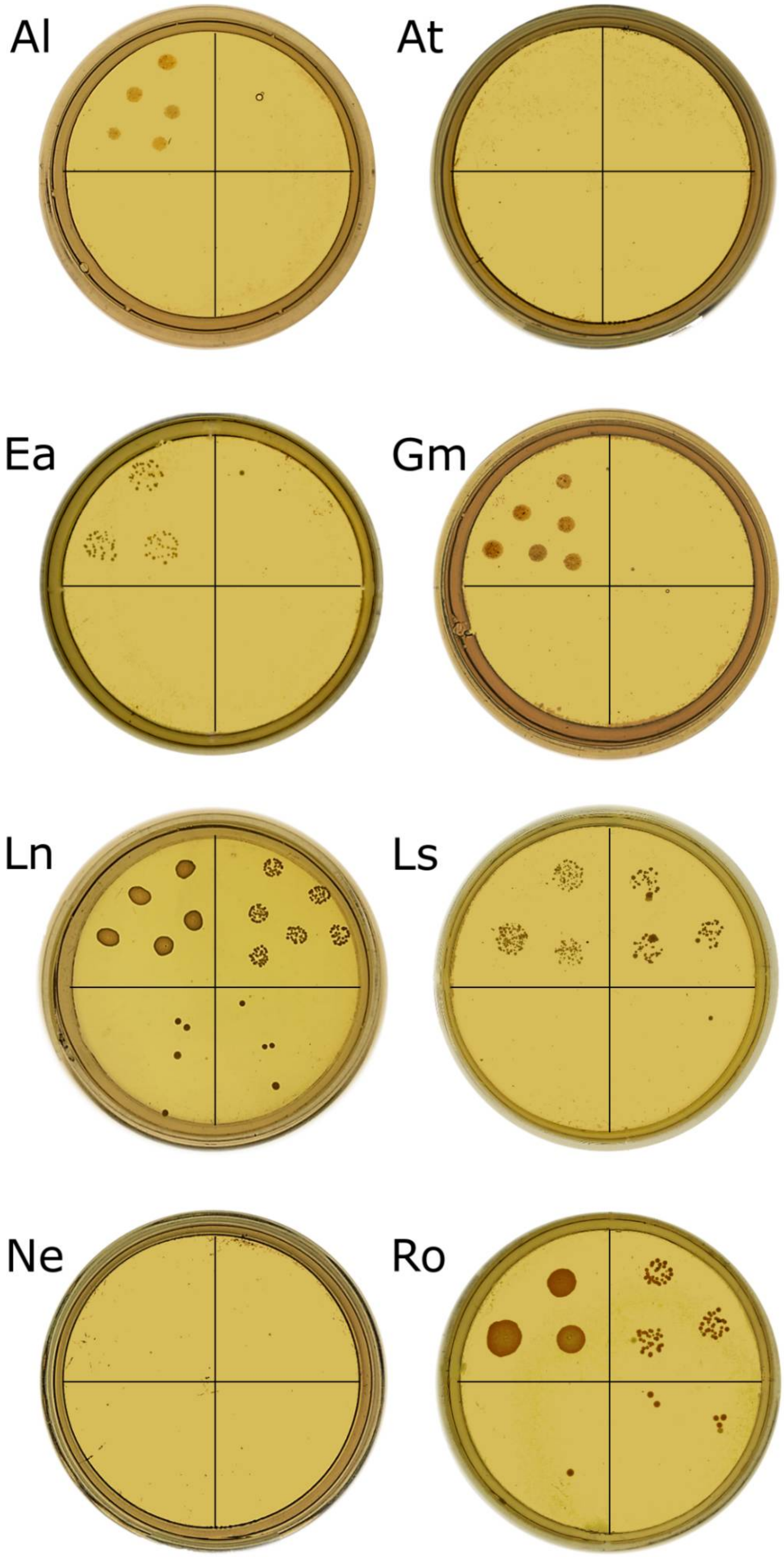

Supplement: Supplementary file 1 [file antibiotics-11-01557-s001.zip › antibiotics-1998629-supplementary.pdf]
